# Bioinformatics Modelling and Metabolic Engineering of the Branched Chain Amino Acid Pathway for Specific Production of Mycosubtilin Isoforms in *Bacillus subtilis*

**DOI:** 10.3390/metabo12020107

**Published:** 2022-01-24

**Authors:** Jean-Sébastien Guez, Françoise Coucheney, Joany Guy, Max Béchet, Pierre Fontanille, Nour-Eddine Chihib, Joachim Niehren, François Coutte, Philippe Jacques

**Affiliations:** 1 Institut Pascal, Clermont Auvergne INP, CNRS, Université Clermont Auvergne, F-63000 Clermont-Ferrand, France; j-sebastien.guez@uca.fr (J.-S.G.); pierre.fontanille@uca.fr (P.F.); 2 Équipe Métabolites Secondaires d’Origine Microbienne, Institut Charles Viollette, UMRt BioEcoAgro 1158-INRAE, Université de Lille, F-59000 Lille, France; francoise.coucheney@univ-lille.fr (F.C.); guy.joany@gmail.com (J.G.); max.bechet@univ-lille.fr (M.B.); 3 UMR 8207–UMET–Unité Matériaux et Transformations, Centrale Lille, INRAE, CNRS, Université de Lille, F-59000 Lille, France; nour-eddine.chihib@univ-lille.fr; 4 Biocomputing Team, Centre de Recherche en Informatique, Signal et Automatique de Lille CRIStAL, UMR CNRS 9189, Université de Lille, F-59000 Lille, France; joachim.niehren@univ-lille.fr; 5 INRIA, Université de Lille, F-59000 Lille, France; 6 Équipe Métabolites Spécialisés d’Origine Microbienne, UMRt BioEcoAgro 1158-INRAE, TERRA Teaching and Research Centre, MiPI, Gembloux Agro-Bio Tech, Université de Liège, B-5030 Gembloux, Belgium; philippe.jacques@uliege.be

**Keywords:** metabolic engineering, bioinformatic modelling, lipopeptides, mycosubtilin, antifungal, fatty acids, branched chain amino acids, *Bacillus subtilis*

## Abstract

Mycosubtilin belongs to the family of lipopeptides. Different isoforms with various antifungal activities can be obtained according to the length and the isomery of the fatty acid. In this work, the activities of the mycosubtilin isoforms were first studied against the pathogen *Aspergillus niger*, revealing the high activity of the *anteiso*-C17 isoform. Modification of the mycosubtilin isoform patterns during cultures of the natural strain *Bacillus subtilis* ATCC 6633 was then investigated through amino acid feeding experiments. In parallel, single-gene knockouts and single-gene overexpression, leading to the overproduction of the *anteiso*-C15 fatty acid chains, were predicted using informatics tools which provide logical reasoning with formal models of reaction networks. In this way, it was *in silico* predicted that the single overexpression of the *ilvA* gene as well as the single knockout of the *codY* gene may lead to the overproduction of *anteiso*-C15 fatty acid chains. For the first time, it has been demonstrated that overexpression of *ilvA* helps to enhance the furniture of odd *anteiso* fatty acids leading to a favored mycosubtilin *anteiso*-C17 production pattern (+41%). Alternatively, a knock-out *codY* mutant led to a higher furniture of even *iso* fatty acids, leading to a favored mycosubtilin *iso*-C16 production pattern (+180%). These results showed that increased selective synthesis of particular isoforms of mycosubtilin through metabolic engineering is feasible, disclosing the interest of these approaches for future development of lipopeptide-producing strains.

## 1. Introduction

**Figure 1:**
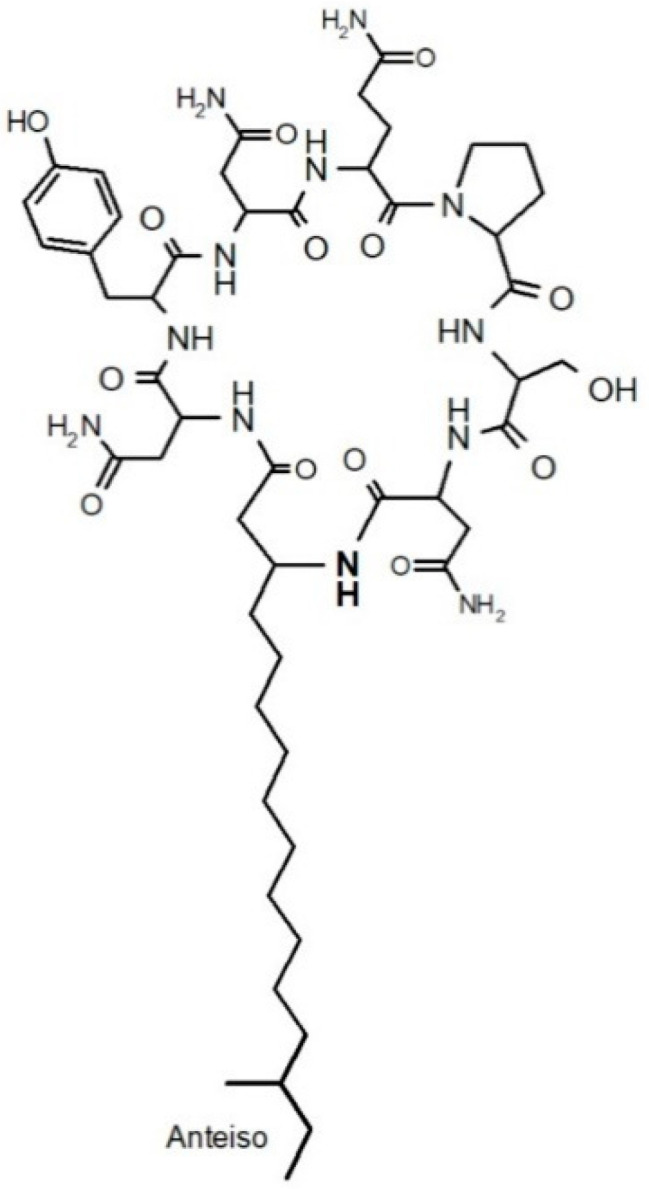
Mycosubtilin *anteiso*-C17.

Lipopeptides are classified into three families: surfactins, fengycins (or plipastatins), and iturins [1]. Among all these natural products, mycosubtilin is a hybrid polyketide synthase/nonribosomal peptide synthase (PKS/NRPS) product which belongs to the iturin family. This family also includes iturin A, A_L_, and C; bacillomycin D, F, L, and L_C_; and bacillopeptin [2]. Mycosubtilin is a biosurfactant and a strong antifungal agent constituted of a circular lipoheptapeptide with a *β*-amino fatty acid moiety (Figure 1).

The combination of different fatty acid lengths, mainly C16 and C17, and multiple isomeries (linear, *iso*, and *anteiso*) leads to different mycosubtilin isoforms, showing varying biological activity. Mycosubtilins act as pore-forming agents that activate *in situ* phospholipases and allow entering and disrupting the lipid bilayer by forming oligomers. These oligomers consist of lipopeptide clusters, lipopeptide–phospholipids, or phospholipid–sterols complexes [3]. The pores formed will gradually expand and then lead to the death of the target cell through the leakage of potassium, intracellular nucleotides, proteins, lipids, and polysaccharides [4,5]. The pore-forming mycosubtilin shows a wide antifungal spectrum against *Saccharomyces cerevisiae* [6], *Candida albicans*, *Yarrowia lipolytica*, *Pichia pastoris*, *Cryptococcus neoformans* [7], *Candida krusei*, *Paecilomyces variotii*, *Byssocchlamys fulva* [8], *Botrytis cinerea* [9], *Venturia inaequalis* [10], *Zymoseptoria tritici* [11], and *Bremia lactucae* [12]. However, all of these studies have been performed with mixtures of mycosubtilin isoforms produced by *B. subtilis*.

It has long been known that the composition of the peptide chain has an influence on the antifungal activity of iturins [6,13]. To illustrate the significance of the relationship between specific primary structures and related biological activities [14], two-dimensional NMR studies were performed with mycosubtilin [15] and iturin A [16]. Despite their close-related primary peptidic structures which only differ by the inversion of two residues in positions 6 and 7, D-Ser and L-Asn, these two compounds showed a completely different molecular topology, resulting in different levels of biological activity. The impact of both the length of the fatty acid chain and its isomery seems to be even more important than that of the core peptide. Thus, the surfactant activity of iturins and the stability of the foam is impacted by the length of the alkyl chain. Increasing the chain length (above C15) results in lower bubble stability [17]. The nature of the fatty acid chain also plays an important role in the antifungal activity of iturins. A study carried out on the effect of the chain’s length and the isomery of iturin A showed that the antifungal activity was even higher when the chain was longer [18]. It also appeared that the iturin A linear *n*-C16 isoform was more active than the *iso*-C16 isoform. Another study on iturins showed that the *anteiso*-C17 isoform of mycosubtilin was the most active against the yeast *C. albicans* [7]. The activity of different alkyl chains of mycosubtilin against *B. cinerea* was also studied. The minimal inhibitory concentration (MIC) of purified isoforms of mycosubtilin was determined and the results showed that the *anteiso*-C17 isoform was the most active against *B. cinerea* with a MIC of 8 μM, followed by the isoforms *n*-C16 and *iso*-C17 (MIC = 16 μM) and *iso*-C16 (MIC = 32 μM) [9]. Moreover, bacillomycin L (and Lc) showed that the antifungal activity increased with the length of the alkyl chain *n*-C16 ≥ *n*-C14 and *iso*-C16 ≥ *iso*-C14. Furthermore, in this same study, the authors showed that the isomery was also important as *n*-C16 was more active than *iso*-C16 [19].

To better understand how *B. subtilis* produces the different mycosubtilin isoforms, the synthesis mechanism of mycosubtilins, which helps to incorporate the fatty acid moiety, should be highlighted. Mycosubtilin synthesis is accomplished by the mycosubtilin synthetase, a multifunctional hybrid type I PK synthetase/NRP synthetase (PKS/NRPS). The operon encoding the mycosubtilin synthetase has been identified and sequenced in *B. subtilis* ATCC 6633 [20]. This operon of 38 kb consists of four ORFs: *fenF*, *mycA*, *mycB*, and *mycC*. The assembly line of the mycosubtilin synthetase directs the mycosubtilin formation through a complex thiotemplate modular system. Each of the seven NRPS modules of the mycosubtilin synthetase, distributed among MycA, MycB, and MycC subunits, are responsible for the incorporation of a single amino acid. MycA presents unique features with combining functional fatty-acid-activating PKS and NRPS modules. An intra-cellular fatty acid is loaded by an acyl ligase (AL) domain onto acyl carrier protein (ACP1). The loading of the acyl by (AL) does not depend on the length of carbon chain when it is included between C10 and C16 [21]. Moreover, the lack of specificity of AL, FenF, and also AMT [22] opens interesting combinatorial biosynthesis prospects for the development of more active analogues [23]. However, no data are available yet on the *in vivo* specific loading of ACP1 by AL with natural *iso*- or *anteiso*-branched fatty acids of *B. subtilis*. The synthesis mechanism of the different mycosubtilin isoforms could, thus, depend on the intra-cellular fatty acid pool. Particularly, the synthesis of *anteiso*-branched chain mycosubtilin, which seems to be the most antifungal isoform [7], could depend on the *anteiso*-branched chain fatty acids pool. 

So far, the branched chain fatty acids in bacteria belonging to *Bacillus* genus are *de novo* synthesized [24] using the branched chain α-keto acids coming from valine, leucine, and isoleucine (Val, Leu, and Ile) as primer sources of even *iso*, odd *iso*, odd *anteiso* fatty acids, and malonyl-CoA as the chain extender [25]. *B. subtilis* possesses branched chain fatty acids of the *iso* and *anteiso* series as major acyl constituents of cellular lipids: odd *anteiso* (43%), odd *iso* (29%), and even *iso* (15%). The branched chain amino acids (Val, Leu, and Ile) are biosynthesized by several enzymes encoded by the gene *ilvA* and those of the *ilvB* operon using threonine and pyruvate as precursors [26,27]. More specifically, the *ilvA* gene codes for the threonine dehydratase, which catalyzes the first step of the Ile biosynthesis pathway [28]. During the biosynthesis of branched chain fatty acid, these amino acids are first degraded to α-keto-3-methylvalerate (Keta), α-ketoisovalerate (Ketb), and α-ketoisocaproate (Ketc) by a branched chain amino acid transaminase (BCAT). These intermediates are then converted to 2-methylbutyryl-CoA (MetButCoA), isobutyryl-CoA (IsoButCoA), and isovaleryl-CoA (IsoValCoA), respectively, by the branched chain ketoacid dehydrogenase (BCKD) complex. These two groups of enzymes (BCAT and BCKD) are encoded by the *bkd* operon (*ptb, bcd, buk, lpdV, bkdAA, bkdAB*, *and bkdB*) [29]. This metabolic pathway is regulated by the three major transcriptomic regulators of *B. subtilis*, CodY (the global regulator), TnrA (the nitrogen regulator), and CcpA (the carbon-activated protein) [30]. The importance of this metabolic pathway in the production of *Bacillus* lipopeptides has recently been studied. Coutte et al. (2015) has modelled this metabolic pathway and its regulation in order to successfully predict the gene knock-out for the overproduction of Leu and *in fine* surfactin [27]. More recently, the same team has shown that by modifying the catabolic pathways of branched chain amino acids, and particularly by disrupting the *lpdV* gene, a strain derived from *B. subtilis* 168 preferentially produced *n*-C14 surfactin [31], while an interruption of *codY* caused an overproduction of surfactin and particularly of the [Val]7 C14 isoform. Other authors have shown that the inhibition of *bkdAA* and *bkdAB*, two genes involved in the degradation of Leu and Val to fatty acids, not only improved surfactin production, but also increased the proportion of C14 isoforms. The mutant strain produced nearly 85% of this isoform compared to 25% obtained with the parent strain [32]. Regarding the production of iturinic molecules, it has been shown, using *B. subtilis* BD, BL, MS, IT, and NICIB 8872 strains, that supplying the culture medium with exogenous Val, Leu, and Ile subsequently orientated the fatty acid synthesis toward even *iso*, odd *iso*, and odd *anteiso* fatty acids, and favored the production of even *iso*, odd *iso*, and odd *anteiso* lipopeptides [33]. These elements infer that the production pattern of mycosubtilin isoforms could be modulated by targeting genes that modify the intracellular branched fatty acid pool.

In this study, we first investigated the activities of the different mycosubtilin isoforms against the pathogen *Aspergillus niger*, revealing the high activity of the *anteiso*-C17 isoform. As branched chain amino acids are primer sources of branched chain fatty acids, we compared then the production of mycosubtilin isoforms by *B. subtilis* ATCC 6633 with or without exogenous branched amino acid feeding. Finally, we developed parallel approaches based on transcriptomic studies, as well as bioinformatic modelling and prediction, to identify several target genes which could lead to the production of different patterns of mycosubtilin. Evidence of the role of one chosen target gene in the enhanced furniture of the fatty acid isoform was achieved, leading to a favored bioactive mycosubtilin production pattern. 

## 2. Results

The objectives of this work were first to carry out the purification of mycosubtilin isoforms to investigate their antifungal activity against *A. niger,* which causes major problems in fruit and vegetable crops, particularly in the cultivation of *Amaryllidaceae*. Once the most active isoform was identified, different overproduction strategies were implemented, such as amino acid feeding, gene knock-out, or gene overexpression, to direct the *Bacillus*’s metabolism through the production of this most active isoform.

### 2.1. Purification and Determination of the Antifungal Activity of Mycosubtilin Isoforms

Mycosubtilin isoforms were produced by the *B. subtilis* BBG100 strain, using the overflowing continuous culture (O-CC) process described by Guez et al. (2021) [34]. Figure S1 presents the annotated chromatogram obtained for a mixture of mycosubtilins composed of: *iso*-C16 (26%), Gln3 C17 (1%), *n*-C16 (2%), *anteiso*-C17 (45%), and *iso*-C17 (23%). This mixture was used for the chromatographic preparation of each individual mycosubtilin isoform. MICs of the mixture and of each purified isoform were then determined against *A. niger*, and compared with previously obtained results toward various fungi (Table 1).

Confirming the results previously obtained by authors on the ascomycetes fungi *B. cinerea*, the solution of mycosubtilin had a strong action against *A. niger*. The minimal inhibitory concentrations of the mixture of mycosubtilins and individual purified mycosubtilin isoforms showed that the mixture gave an equal effect compared to the individual purified mycosubtilin isoforms. Indeed, the *anteiso*-C17 mycosubtilin and the mix were the most active against the fungus (MIC of 8 µM each), followed by the *n*-C16 and *iso*-C17 isoforms (MIC of 16 µM each), as well as the *iso*-C16 one (MIC of 32 µM). The *anteiso*-C17 mycosubtilin seems significantly less active against *C. albicans* (32 µM). Taken together, these results show that it could, therefore, be interesting to overproduce this *anteiso*-C17 isoform specifically, in order to facilitate the purification step and the potential future use of this compound as an antifungal agent.

### 2.2. Effect of Amino Acid Feeding during B. subtilis ATCC 6633 Cultures

#### 2.2.1. Effect on Cellular Fatty Acids Pattern

**Figure 2:**
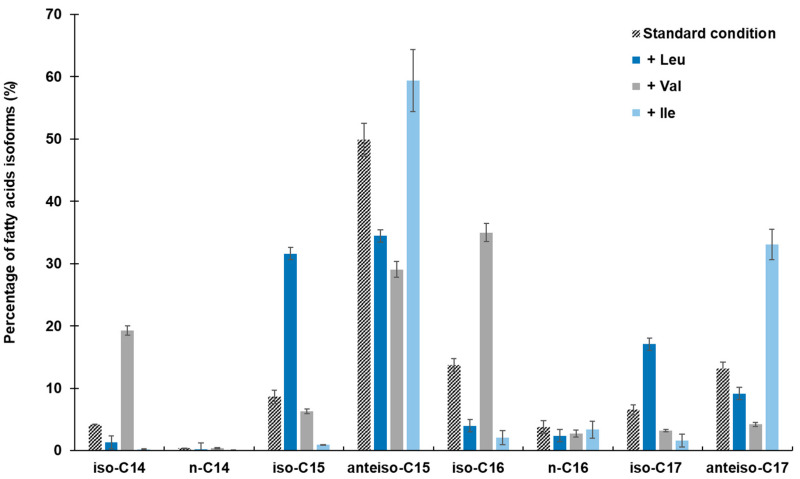
Fatty acid isoforms pattern of *B. subtilis* ATCC 6633 after 48 h of growth at 30 °C in modified Landy medium buffered at pH 7.0 with MOPS 100 mM and supplied either with Leu, Val, or Ile at 2 g/L. Results are mean values and standard deviations of four independent experiments.

The proportions of the different fatty acid isoforms were quantified by gas chromatography in amino-acid-supplemented conditions (Figure 2). The addition of Leu to the culture medium induced an increase in the odd *iso* fatty acids, such as *iso*-C15, which increased from 8.62 to 31.59% and *iso*-C17 which increased from 6.54 to 17.06% compared to the control. The even *iso* fatty acids show an increase, from 13.72 to 34.98% for *iso*-C16 and 4.16 to 19.26% for *iso*-C14, in the presence of Val. Finally, the addition of Ile leads to an increase in *anteiso* fatty acids, from 49.91 to 59.40% for *anteiso*-C15 and from 13.12 to 33.07% for *anteiso*-C17.

#### 2.2.2. Effect on Mycosubtilin Pattern

**Figure 3:**
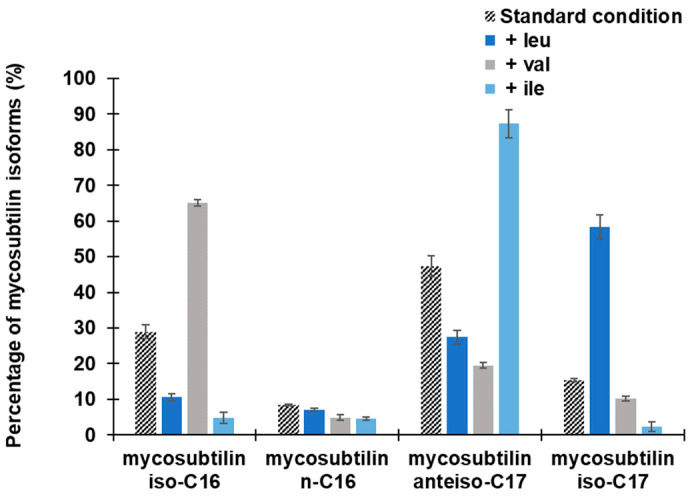
Mycosubtilin isoforms pattern of *B. subtilis* ATCC 6633 after 48 h of growth at 30 °C in modified Landy medium buffered at pH 7.0 with MOPS 100 mM and supplied either with Leu, Val, or Ile at 2 g/L. Results are mean values and standard deviations of four independent experiments.

The mycosubtilin production was measured after 48 h of growth. Mycosubtilin concentration was above 55.0 ± 10.3 mg/L for the standard condition and was slightly higher for the amino acids supplemented conditions: 66.1 ± 5.2 mg/L for Leu, 71.4 ± 5.4 mg/L for Val, and 77.3 ± 7.6 mg/L for Ile. The growth of *B. subtilis* ATCC 6633 was comparable under all the used conditions (data not shown). Supplementation of the modified Landy medium with different amino acids had an impact on the diverse proportions of mycosubtilins patterns (Figure 3). Branched amino acids showed different impacts on isomers production: Val helped to obtain mainly mycosubtilin *iso*-C16, Leu helped to obtain mainly mycosubtilin *iso*-C17, and Ile led to a highly selective *anteiso*-C17 mycosubtilin production pattern.

The ratio of *anteiso* on *iso* fatty acids to mycosubtilin isoforms was calculated. This indicated the very significant effect of the addition of Ile for the biosynthesis of *anteiso* fatty acid as well as its clear impact on the production profiles of mycosubtilin. Indeed, the ratio of FA *anteiso*/*iso* is 19.2 ± 3.4 and the ratio of mycosubtilin *anteiso*/*iso* is 12.3 ± 1.4.

### 2.3. Identify Genetic Targets for Specific Production of Mycosubtilin Isoforms

A main objective of this work was the development of a metabolic engineering-like strategy to direct the mycosubtilin synthesis selectively toward a chosen isoform. To achieve this goal, two parallel and complementary approaches were developed. The first is based on a transcriptomics study of *B. subtilis* BBG100 in the presence or absence of Ile in the medium. The second is based on bioinformatics modelling of the branched chain amino acid metabolic pathway and the use of constraint programming to predict potential genetic targets for overexpression or knockout, leading to an overproduction of the *anteiso*-branched chain fatty acid.

#### 2.3.1. Transcriptomic Study

The differential gene expression occurring during an overproduction of the *anteiso*-C17 mycosubtilin compared to a standard mycosubtilin production pattern was studied. As yeast extract contains branched amino acids, it was retrieved from the modified culture medium. Under these conditions, a loss of productivity in mycosubtilin was observed by a 20-fold decrease in *B. subtilis* ATCC 6633 and a 5-fold decrease with its derivative BBG100 (data not shown). The choice was, therefore, made to preferentially study *B. subtilis* BBG100 transcriptomic response rather than a *B. subtilis* ATCC 6633 one, as *B. subtilis* BBG100 guarantees a level of mycosubtilin production between 10 and 15 times higher than that of ATCC 6633 [35]. The strain BBG100 was constructed from *B. subtilis* ATCC 6633 by replacing the native promoter of the mycosubtilin operon by a strong and constitutive one [35]. Moreover, to intensify the mycosubtilin production, the culture medium was buffered with 100 mM MOPS at pH 7.0 [34] and cultures were grown under conditions of high oxygen limitation [36]. Finally, cultures of *B. subtilis* BBG100 were performed in the culture medium supplemented with 2 g/L of Ile (test) or not (control).

**Figure 4:**
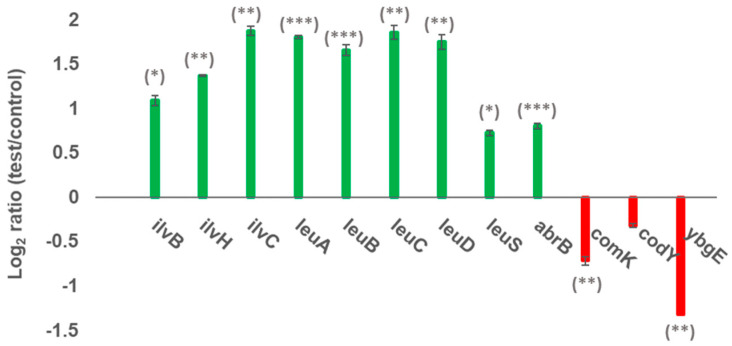
Microarray results for differential gene expression performed in the culture medium supplemented with 2 g/L of Ile (test) or not (control). *p*-value < 0.05: *; *p*-value < 0.01: **; *p*-value < 0.001: ***; result based on a wrapper-based gene selection technique (bootstrap classification combined to variance analysis): *codY*.

Under these experimental conditions, the differential gene expression of the mycosubtilin overproducing strain BBG100 could be inspected. A DNA oligonucleotide microarray analysis revealed differential level of expressions of transcripts involved in central metabolic pathways linked to the selectivity of the *anteiso*-C17 mycosubtilin biosynthesis. Samples taken from cultures for transcriptome studies were collected early in order to gain access to information on genetic regulations occurring early in growth (Figure 4). Data showed that, in the presence of Ile in the medium, most of the early regulated gene and overexpressed genes are involved in the metabolic pathway of branched chain amino acid biosynthesis and with a high expression of the *ilvBHCleuABCD* operon. In contrast, the *ybgE* gene coding for aminotransferase is under-expressed. Furthermore, from a regulatory point of view, the global pleiotropic regulator *codY* is slightly under-expressed during growth in the presence of Ile, whereas *abrB* is overexpressed.

#### 2.3.2. Model-Based Prediction for the *anteiso* Fatty Acid Precursor Overproduction

To perform a bioinformatic prediction of the overproduction of *anteiso*-C17 mycosubtilin, the previously developed model of the branched chain amino acid metabolic pathway was used [27]. This model was developed at the time to predict the genes knock-out and the changes in input flow required for the overproduction of Leu (Figure S2). In the present work, this model was improved by taking advantage from the latest developments in the field. All the changes and simplifications made are described in detail in paragraph 4.4 of the Materials and Methods section. The prediction target of interest for *anteiso*-C17 mycosubtilin production is to increase the outflow to *anteiso*-C15 fatty acid biosynthesis. So, the target is now: *anteiso*-C15 = ↑.

**Figure 5:**
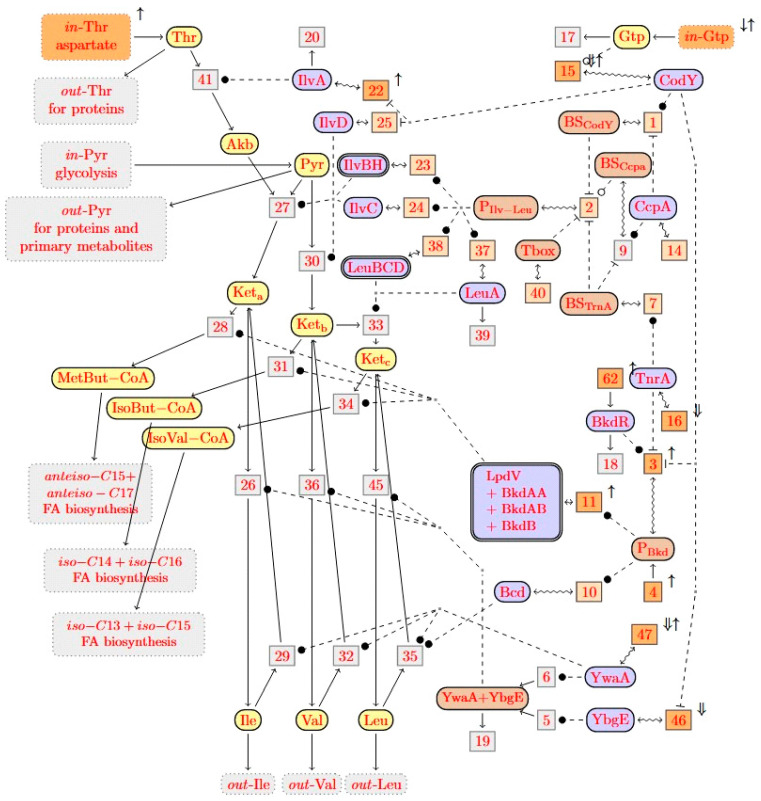
New improved model of branched chain amino acid metabolic pathway developed for the overproduction of mycosubtilin *anteiso*-C17. Constraint was applied on the overproduction of *anteiso*-branched chain fatty acid (*anteiso*-C15 + *anteiso*-C17 = ↑) and change predictions were visualized. Modelling language and semantics were first described by Coutte et al. (2015) [27], and improvements for this work are described in the paragraph 4.4 of the Materials and Methods section. This figure semantic description was extracted from Coutte et al. (2015) [27] for better understanding of this figure. Reaction networks in our modeling language are represented as graphs similar to Petri nets. The concrete syntax of our reaction networks is based on XML, from which the graphs are computed. The XML representation is also the input for the prediction algorithm. These graphs contain two kinds of nodes: round nodes for representing its species and boxed nodes for representing its reactions. More precisely, any species *S* is represented by a round node 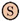 and any reaction with name r by a boxed node 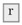. Solid edges either link a reactant to its reaction 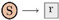 or a reaction to one of its products 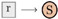. There are three kinds of dashed edges, which start at the three kinds of modifiers. An accelerator edge links an accelerator to a reaction 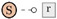, an activator edge links an activator to a reaction 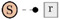, and an inhibitor edge links an inhibitor to a reaction 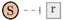. An input edge 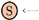 points from the context to an inflow species *S*, while an outflow edge 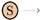 points from an outflow species *S* to the context. For convenience, we introduced the last kind of edges 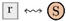 as a shortcut for a product that is degraded by a hidden reaction, i.e., as a shortcut for 
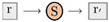
. Species nodes with three different colors were used, which indicate their biological roles. Yellow indicates metabolites (such as 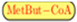) and blue indicates proteins (such as 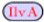). There is a third color for “artificial species” that serves to modulate regulation, such as the promoter of the *ilv-leu* operon 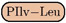. Reactions that are potential candidates for knockouts or overexpression will be annotated in orange. Dark orange indicates candidates that were selected by our knockout prediction, while light orange indicates candidates that were not. Genes knockout predictions are represented by ⇓ and gene overexpression by ↑.

Running the version 0.6 of the BioComputing’s reaction network tool with this target on the reaction network did not work well the first time, for several reasons that are described in detail in the Materials and Methods section, which led to the refined feedback network presented in Figure S4. Running the BioComputing’s tool again on the reaction network from Figure S4 led to much more convincing predictions of reaction knockouts, inflow changes, and gene overexpression, as described in Figure 5.

First predictions are linked to input flows, i.e., an increase in the input flow of Thr and GTP is proposed. Second predictions propose the knock-out of several genes, in particular those involved in reactions r15 (CodY biosynthesis), r16 (TnrA biosynthesis), r46 (YbgE biosynthesis), and r47 (YwvA biosynthesis). Last predictions are linked to the overexpression of genes and particularly for the reactions of r22 (IlvA biosynthesis), r3, r4, r62 (expression of *bcd-bkdL* operon), and r47 (YwvA biosynthesis). Surprisingly, some reactions are predicted in Up and Down; this is the case of reactions r15 (CodY biosynthesis) and r46 (Ywva biosynthesis). All these predictions are discussed below. The two criteria were followed to choose the target genes: (1) to choose a reaction directly impacting the metabolic reactions; and (2) to choose of a reaction impacting the regulation of these metabolic pathways. Thus, the overexpression of the *ilvA* gene (r22) and the interruption of the *codY* gene (r15) were undertaken by genetic engineering to verify their impact on the production of *anteiso* fatty acid and mycosubtilin *anteiso*-C17.

### 2.4. Genetic Engineering of Predicted Gene Targets

**Figure 6:**
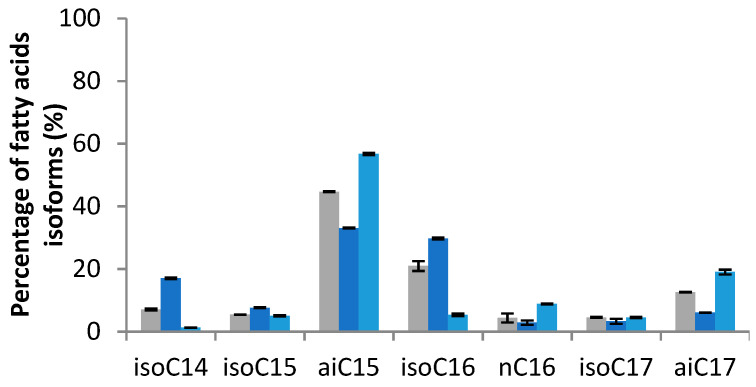
Fatty acid isoforms profiles of *B. subtilis* ATCC 6633 (gray bar), BV12I37 (CodY−; dark blue bar), and BBG133 (IlvA+; light blue bar) after 48 h of growth at 30 °C in the modified Landy medium. Results are mean values and standard deviations of two independent experiments.

**Figure 7:**
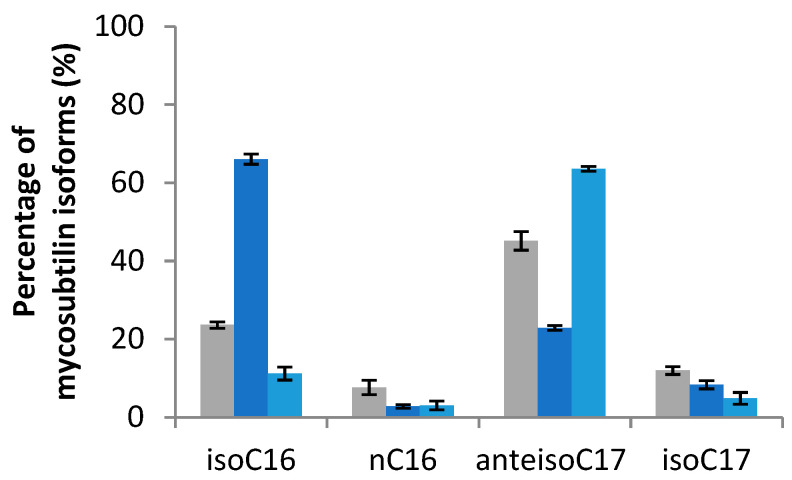
Patterns of mycosubtilin isoforms of *B. subtilis* ATCC 6633 (gray bar), BV12I37 (CodY−; dark blue bar), and BBG133 (IlvA +; light blue bar) after 48 h of growth at 30 °C in the modified Landy medium. Results are mean values and standard deviations of two independent experiments.

From the above results, several genetic targets were identified as having a major role in the modulation of the production of mycosubtilin isoforms. The overexpression of *ilvA* (BBG133) and the knock-out of *codY* (BV12I37) were performed in the wild-type strain. These strains were then cultured at 30 °C in modified Landy medium for 48 h. The fatty acid isoforms were quantified by GC and the mycosubtilin concentration, as well as isoforms by HPLC. The strains *B. subtilis* ATCC 6633 and BBG133 showed similar growths, compared to BV12I37, which was slightly lowered (data not shown). The mycosubtilin production measured after 48 h of growth was 55.0 ± 10.3 mg/L for *B. subtilis* ATCC 6633, 45.7 ± 1.3 mg/L for BV12I37, and 36.7 ± 18.1 mg/L for BBG133. The diverse isoform proportions of fatty acids were quantified by GC (Figure 6) and the mycosubtilin profile was quantified by HPLC (Figure 7).

The membrane fatty acids pattern of the *B. subtilis* BBG133 performed at 30 °C was widely dominated by odd-numbered branched chains in comparison with the profile of the wild-type ATCC 6633. The predominant fatty acid was *anteiso*-C15, whereas the *anteiso*-C17 and the *iso*-C16 were, respectively, the second and third common fatty acids. The main changes were observed for the *anteiso*-C15 whose relative amount raised from 44.6% to 56.7%, respectively, for ATCC 6633 to BBG133. Moreover, the results showed a selective *anteiso*-C17 mycosubtilin production pattern, and that the *anteiso*-C17 relative amount increased from 45.15% for ATCC 6633 to 63.6% for BBG133. The *iso*-C16 isoform represented only 11.7%.

In the mutant CodY−, fatty acid profiles were more directed on even-numbered branched chains. In fact, the *anteiso*-C15 and *iso*-C16 are equivalently proportional at 33.0% and 29.7%, respectively, and the third most common fatty acid is *iso*-C14, whose relative amount reaches 17% but represents just 7% for ATCC 6633 profile. The mycosubtilin profile is thus directed toward a selective *iso*-C16 (66.1%) pattern.

## 3. Discussion

### 3.1. Purification and Determination of the Antifungal Activity of Mycosubtilin Isoforms

Mycosubtilin is a lipopeptide with a large antifungal spectrum against phytopathogens, foodborne pathogens, and food spoilage [1,2,8]. Its antifungal activity is the most important among the iturinic lipopeptides, such as bacillomycin L, bacillomycin F, bacillomycin D, and iturin A [37]. The different mycosubtilin isoforms, composed of different fatty acid length and multiple isomery, presented various biological activities, selective antifungal activities, or biosurfactant activities. Many studies were performed with mixtures of mycosubtilin isoforms produced by *B. subtilis*. In this work, each major mycosubtilin isoform was purified in order to test its antifungal activity on a food spoilage microorganism, *A. niger*.

Our findings showed that the *anteiso*-C17 mycosubtilin is the most interesting isoform due to its high activity (MIC = 8 μM) against *A. niger*, at the same level as against *B. cinerea* [9]. Its activity is even higher than the one of the *iso*-C17 mycosubtilin (MIC = 16 µM), showing the importance of the isomery of the fatty acid moiety. Another result is that the higher activity of mycosubtilin *iso*-C17 compared to the one of the *iso*-C16 isoform is in line with the study carried out previously on iturin A, showing that the antifungal activity is even higher when the chain is longer [18]. The differences of MIC values obtained for *A. niger* compared to those previously obtained for *C. albicans* could be explained by the main differences in the composition of the membrane composition of these microorganisms [38,39].

### 3.2. Effect of Amino Acid Feeding during B. subtilis ATCC 6633 Cultures

In order to increase the biosynthesis of one specific mycosubtilin isoform, a first approach of feeding experiments with branched chain amino acids, i.e., precursors of fatty acids, was performed.

The results presented in Figure 2; Figure 3 show that the supply in Val favors the biosynthesis of even fatty acid, which then results in a high production of mycosubtilin C16 isoform. In contrast, Leu and Ile lead to an increase in odd fatty acids (C15 and C17), specially the *anteis*o form for the supply with Ile and, thus, a highly selective *anteiso*-C17 mycosubtilin production pattern. Ile is the precursor of odd-branched fatty acids [25,27]. These results were in accordance with those obtained previously by other authors [7] who used Ile as a nitrogen source for the specific production of *anteiso*-C17 mycosubtilin. Formerly, changes in the proportions of counterparts bacillomycin F and L have been obtained by replacing only the Landy source of nitrogen by other amino acids [33]. Besson et al. studied the proportions of β-hydroxy fatty acid incorporated into a lipopeptidic compound according to different conditions of addition of amino acids (Leu, Ile and Val) in different strains of the *B. subtilis*-producing iturinic compound [33]. They reported that during the biosynthesis of this lipopeptide, the presence of Val could increase the proportion of even *iso* β-hydroxy fatty acid, the presence of Ile of *anteiso* odd β-hydroxy fatty acid (especially counterpart C15), and the presence of Leu of *iso* odd β-hydroxy fatty acid [33]. Similar work was carried out by these authors, based on the incorporation of radioactive sodium acetate into fatty acids, who attempted to elucidate the biosynthesis mechanism in iturin-producing *B. subtilis* [40].

### 3.3. Modification of the Selective Synthesis of anteiso-C17 and iso-C16 Mycosubtilin Isoforms

As shown in this work, the modification of the cellular fatty acid’s patterns observed with *B. subtilis* mutants BBG133 (IlvA+) and BV12I37 (CodY−), compared to the reference strain *B. subtilis* ATCC 6633, helps to modify the mycosubtilin isoform patterns. The *anteiso*-C17 mycosubtilin production increased from 45.1% for ATCC 6633 to 63.6% for BBG133 (IlvA+), whereas the *iso*-C16 mycosubtilin production increased from 23.6% for ATCC 6633 to 66.1% for BV12I37 (CodY−). These results infer that the production pattern of the different mycosubtilin isoforms is directly linked to the cellular fatty acid pool. Previous results showed the in vitro lack of specificity of the fatty acid loading domain (AL) of the mycosubtilin synthetase. Indeed, the loading of the acyl by (AL) does not seem to depend on the length of the carbon chain when it was comprised between C10 and C16 [21]. The present work demonstrates that the *in vivo* loading of fatty acids again shows its tolerance; this time, there was tolerance for the loading of different fatty acid isoforms, including natural *iso* or *anteiso*-branched, which could lead to interesting combinatorial biosynthesis prospects.

### 3.4. Identifying Genetic Targets to Increase the Specific Production of Mycosubtilin Isoforms

The biosynthetic pathways of branched chain amino acid (BCAA) in *B. subtilis* are well known and previously modelized [27]. The genetic loci involved are mainly *ilvBHCleuABCD*, *ilvA*, *ilvD*, *ybgE*, and *ywaA* [26]. All of these genes or operons are repressed under amino-acid-rich conditions by the activity of the pleiotropic transcriptional regulator CodY [41,42,43]. Val and Ile are positive effectors of CodY, such that BCAA biosynthesis is negatively autoregulated at relatively low BCAA concentrations [41]. In *B. subtilis*, CodY controls the expression of a several transcriptional units. The presence of Ile, Val, and Leu in the growth medium is essential to obtain both high activity and efficient regulation of target genes [44]. This metabolic pathway is at the heart of the production of branched chain amino acids, but also of branched chain fatty acids, and is therefore of great interest when one wishes to modify the specific production of lipopeptide isoforms.

In this work, a complementary approach was used to identify potential gene target to be modified in order to produce more specifically different mycosubtilin isoforms, i.e., *anteiso*-C17 and also *iso*-C16. This approach combined a transcriptomic study in the presence of Ile (an amino acid well identified as a precursor of *anteiso* fatty acids) and a bioinformatics prediction using a previously developed model of the BCAA pathway [27,45]. The results of this approach allowed us to identify several targets genes.

The results of microarray presented in Figure 4 show that in the presence of Ile in the medium, the *ilvBHCleuABCD* is the main operon overexpressed. Nevertheless, the gene *ilvA* which is also directly involved in the metabolic pathway of Ile production, did not show up in the transcriptomic analysis. This result seems quite coherent because this analysis was carried out in the presence of an excess of Ile in the medium, so that the cells seem to counterbalance their metabolism towards the production of Leu and Val [41]. The addition of Ile in the culture medium led to the repression of *ybgE*, which codes for an aminotransferase involved in the BCAA metabolism. Its repression should physiologically result in the limitation of the synthesis of BCAA. As the growth of the microorganism was normal in the presence of Ile, it was assumed that the cellular pool of Leu and Val needed for cellular growth was not affected. A hypothesis to explain this phenomenon is that the cell could counterbalance the *ybgE* repression by overexpressing the *ilvBHCleuABCD* operon and the *leuS* gene, as shown in the results section of this work. It should be noticed that *ywaA*, which is an isologue of *ybgE* [46], is not repressed in the presence of Ile, which is not critical because of the low enzymatic activity of YwaA compared to YbgE [47].

It is known that several regulators (AbrB and CodY) and environmental factors are implied in the biosynthesis of lipopeptides. The transcriptomic results show that, in the presence of Ile, the *codY* gene is slightly under-expressed (Figure 4). The under-expression of *codY* in the presence of BCAA in the medium was effectively shown by authors [41,42]. The over-expression of the transcriptomic regulator AbrB, which is known to negatively regulate the expression of the mycosubtilin operon [48], was also observed.

In this work, the over-expression of *abrB* was observed, while *comK* was repressed. This concomitant differential expression is consistent with previous results. It was indeed shown that the binding of AbrB at position −35 of the *comK* promoter repressed its expression [49]. This result is also consistent with those obtained in the presence of casamino acids [50]. The concomitant over-expression of *abrB* and under-expression of *codY* is more difficult to explain. Indeed, CodY and AbrB normally act together, for example to repress the expression of *comK* [49]. Our results suggest a reasonably complex mechanism that fine-tunes and regulates the metabolic activity of *B. subtilis* ATCC 6633 when grown in the presence of Ile to promote the synthesis of mycosubtilin *anteiso*-C17.

Prediction results from bioinformatics modelling revealed numerous targets for genetic optimization and specific mycosubtilin isoform production, as presented in Figure 5. Among these, the overproduction of the *ilvA* gene (r22) was predicted. This prediction was then tested *in vivo* by genetically modifying *B. subtilis* ATCC 6633. The overexpression of the *ilvA* gene strengthens the conversion of Thr to AkB by reaction 41. Concomitantly, the outflow of Thr (out-thr) was weakened. If this outflow was absent, all Thr would be converted to AkB in any steady state, so strengthening r41 would have only a short time effect, but not effect in the steady states. Hence, the prediction r22 = ↑ would not be made. This shows that adding the outflow of Thr to the model was indeed relevant for the predictions. As shown in Figure 7, the overexpression of *ilvA* had the expected effects on the specific production of the *anteiso*-C17 mycosubtilin isoform.

From a bioinformatics point of view, it was also verified that all predictions were exact in the sense of [51], which intuitively means that the global reasoning on metabolic loops computes the abstraction perfectly without any over-approximation. It is closely related to the notion of completeness of abstract interpretation. To obtain the most precise result, a recent algorithm for exact prediction was added into the biocomputing reaction network tool developed previously [27,50].

Analysis of the predictions reveal that some reactions are both predicted for single overexpression and single knockouts, for instance reaction r15 (CodY biosynthesis) and r47 (YwvA biosynthesis). This means that they have both a positive and a negative effect on the target. One cannot know which effect is dominating without additional knowledge on the kinetics of the reactions. This double prediction is specifically explained by the multiple involvement of CodY in this complex metabolic pathway [41]. *codY* deletion was also undertaken *in vivo*. This time, the result obtained is not the one expected, and a significant change in the mycosubtilin pattern is indeed observed after the deletion of *codY*, but the isoform *iso*-C16 is favored. This result can be explained by several reasons. First of all, in this metabolic pathway, the deletion of *codY* will certainly lead to a change in the expression of *ilvA* (r22), *ilvD* (r25) genes, *ilv-leu* operon (r2), and the *bkd* operon (r11), but also in the expression of *ybgE* (r46). The analysis of the bioinformatic model helps to explain this result by listing the paths in the graph on which a *codY* knockout can influence the synthesis of the *anteiso*-C15 + C17 fatty acid and also the *iso*-C14 + C16 fatty acid. Experiments showed that overexpression of the *ilvA* gene (r22 = Up) is positive for the target *anteiso*-C15 + C17 fatty acid synthesis. However, the effects on r11 = Up (*bkd* operon) in practice are not known. In the model, the *bkd* operon has a positive influence on both *anteiso*-C15 + C17 fatty acid synthesis and *iso*-C14 + C16 fatty acid synthesis, but these two effects cannot be distinguished. The effect of r46 = Up (*ybgE*) is not clearly defined. In the model, it has a negative effect on both the synthesis of *anteiso*-C15 + C17 fatty acids and the synthesis of *iso*-C14 + C16 fatty acids. From a metabolic point of view, it seems certain that the effect of *codY* deletion leads to the overproduction of the Leu and Val amino acids, as expected. This is the same result observed by Brinsmade et al. (2010) [30] where the deletion of *codY* led to an overproduction of Val by a factor of 6. These amino acids will then be transformed preferably into *iso*-branched fatty acids and, in particular, into even-numbered fatty acids coming from the degradation of Val. This result is consistent with the one obtained on the production of surfactin, where the deletion of *codY* favored the biosynthesis of the surfactin Val7 C14 isoform [31]. Other predictions that were made included the knock-out of *tnrA* and *ybgE*. The deletion of an important regulator, such as TnrA, was expected by the known negative impact of TnrA on the promoter of the *ilv-leu* operon, but also on the promoter of the *bkd* operon [29,52]. It was notably shown that the expression of the *bkd* operon was three times higher in a mutant deleted for *tnrA*. It should be noted that the *tnrA* target did not emerge from the microarray analysis in the presence of Ile, which confirms the result obtained by Debarbouille et al. (1999) who did not observe any change in the expression of this gene in the presence of 20 mM Ile [29]. The modulation of *ybgE* expression is also an interesting target because this gene also emerges from the microarray analysis. Moreover, Cai et al. (2020) also showed that BCAA supply modules may play a role in the production of bacitracin in *B. licheniformis* [53]. In this bacterium, BCAA synthesis pathways were enhanced by the simultaneous overexpression of the acetolactate synthase IlvBNfbr, the 2-isopropylmalate synthase LeuAfbr, and the BCAA aminotransferase YbgE. In addition, permeases were identified as importers of BCAA, and their overexpression enhanced intracellular BCAA accumulations and bacitracin yields [53]. This approach to BCAA-specific permeases would also be interesting to conduct in *B. subtilis* ATCC 6633 with the objective of improving the production of mycosubtilin isoforms.

Concerning the other predictions and, in particular, the overexpression of the *bkd* operon (r3, r4, and r62) and the *ywvA* gene, these predictions seem logical because these genes code for enzymes are directly involved in the degradation of BCAAs and, thus, in the biosynthesis of branched chain fatty acids. However, in a recent study on surfactin, the authors showed that overexpression of the *bkd* operon led to a negative impact on the growth of the strain but also to a significant reduction in surfactin production. Interestingly, this genetic modification was coupled with others, such as the overexpression of the *ilv-leu* operon and the *lipALM* operon (lipoic acid biosynthesis) [54]. This highlights the value of multiple genetic modifications in metabolic engineering strategies.

In view of these known effects of modifying these single targets, it would be appropriate to try a multiple knock-out or mixed knock-out strategy, as well as an overexpression strategy, as has already been undertaken, for example, to increase surfactin synthesis in *B. subtilis* [54]. From a bioinformatics point of view, we also checked that there are no double changes that could satisfy the target containing no predicted single changes. Nevertheless, multiple changes may be reasonable, as the effects may add up. In addition, it may be useful to perform knockouts that disable feedback loops, as one cannot infer from the model what their effect might be without additional kinetic information.

## 4. Materials and Methods

### 4.1. Strains and Growth Conditions

*B. subtilis* ATCC 6633 is a natural isolate producing two lipopeptides, i.e., mycosubtilin and surfactin. All the *B. subtilis* strains used in this study were derivatives from *B. subtilis* ATCC 6633 (Table 2). All strains were stored at −80 °C in 40% (*v*/*v*) glycerol.

The preculture was completed in a modified Clark medium [55], composed as follows: glucose, 20 g/L; KH_2_PO_4_, 2.7 g/L; K_2_HPO_4_, 18.9 g/L; yeast extract, 0.5 g/L; EDTA, 0.05 g/L; MgSO_4_, 0.61 g/L; MnSO_4_, 0.056 g/L; NaCl, 0.1 g/L; CaCl_2_, 0.012 g/L; ZnSO_4_, 0.018 g/L; FeSO_4_, 0.018 g/L; CuSO_4_, 0.002 g/L; Na_2_MoO_4_, 0.001 g/L; H_3_BO_3_, 0.001 g/L; Na_2_SO_3_, 0.001 g/L; NiCl_2_, 0.0037 g/L; NH_4_NO_3_, 4 g/L; and MgSO_4_, 1 g/L. Cells were then transferred to conical flasks of 500 mL at an initial biomass concentration of 0.08 g(D.W)/L, corresponding to an optical density at 600 nm of 0.25. The filling volume of culture medium in the conical flasks was 100 mL. By using this filling volume ratio (0.2), at a shaking speed of 160 rpm and a shaking diameter of 50 mm, the oxygen transfer conditions were limited to 8 mmol O_2_/L/h, allowing an optimization of the mycosubtilin productivity, as shown previously by authors [36]. The cells were cultured in Erlenmeyer flasks at 30 °C, at least in triplicate, and mean values and standard deviations were calculated. For the cultures of the different mutants used in this work, a modified Landy medium was composed as follows: glucose, 20 g/L; (NH_4_)_2_SO_4_, 2.3 g/L; K_2_HPO_4_, 1 g/L; MgSO_4_, 0.5 g/L; KCl, 0.5 g/L; CuSO_4_, 1.6 mg/L; Fe_2_(SO_4_)_3_, 1.2 mg/L; MnSO_4_, 0.4 mg/L; and yeast extract, 1 g/L. For amino acids testing and transcriptomic experiments, the previous medium was modified with retrieving the yeast extract. Culture media were buffered with MOPS (3-(N-morpholino)propanesulphonic acid) at 100 mM at pH 7.0 to favor the synthesis of mycosubtilins [34].

### 4.2. Mutants of B. subtilis ATCC 6633 Used in This Study

Three different mutants from the wild-type strain *B. subtilis* ATCC 6633 were constructed or used in this work. The first one, namely *B. subtilis* BBG100, was obtained in a previous study [35] by replacing the promoter of the mycosubtilin synthase *(mycA*) operon by a strong and constitutive one, i.e., *PrepU* originating from the *repU* gene of *Staphylococcus aureus* plasmid pUB110 [56]. The second one, namely *B. subtilis* BVI2I33, contains a deletion of the *codY* gene, the construction of which has already been described [48].

The third one is an overexpressing *ilvA* mutant strain, namely *B. subtilis* BBG133, which was constructed from the mutant RFB102, a competent strain from wild-type *B. subtilis* ATCC 6633 [7], by replacing the native promoter of the *ilvA* gene by a constitutive one *PrepU*. Briefly, part of the *ilvA* gene was amplified by PCR using *ilvA*-fwd and *ilvA*-rev primers (See Table S1); this fragment was then purified and ligated in pGEM-T easy vector (Promega, Madison, WI, USA) generating the plasmid pBG142. This plasmid was then used to transform *E. coli* JM109. The plasmid pBEST501 was digested by *Xba*I and *Pst*I, generating a fragment containing *PrepU-neo*. The plasmid pBG142 was digested by *Bcu*I and *Mph1103*I generating a long fragment of 4267 bp containing the gene *ilvA*. This longer fragment and the fragment *PrepU-neo* from pBEST501 were ligated together using T4 DNA ligase (Takara, Shiga, Japan), leading to a new plasmid pBG154, which was used to transform *E. coli* JM109. The plasmid pBG154 was then amplified using TempliPhi technology (Cytiva, Malborough, MA, USA) prior to its use for the transformation of RFB102. The transformation of RFB102 with pBG154 was performed using natural competence protocol [57]. Transformation experiments with *B. subtilis* RFB102 and pBG154 led to isolation of one sole colony. The resulting strain of *B. subtilis* was named BBG133. Genomic DNAs of both this clone and the wild-type strain were purified. Replacement of the natural promoter by the constitutive promoter *PrepU* associated with the *neo* gene was demonstrated by PCR amplification of genomic DNA with the *PrepU*-fwd and *ilvA2*-rev primers. A 1.9 kb fragment was obtained instead of nothing with the wild type (data not shown). Strains and plasmid used in this study are presented in Table 2.

### 4.3. RNA Isolation, Reverse Transcription and Microarrays

A volume corresponding to 1 × 10^9^ cells of *B. subtilis* ATCC 6633 was promptly sampled and mixed thoroughly with the Ambion Ribopure RNA latter solution (*v*/*v*). The RNA isolation step was performed specifically for this strain, as described by authors [58]. For microarray assays, the reverse transcription and the cDNA labelling were realized following the protocol described previously [59]. Fluorescently labeled probes were then hybridized to 4096 open reading frame (ORF) microarray slides prepared from *B. subtilis* strain 168 (Eurogentec, Belgium). Microarray hybridization was performed with 10 µg of cDNA with a Discovery Ventana (Ventana, Tucson, AZ, USA). Images were analyzed using GenePix Pro 6 (Axon Instruments, Inc., Union City, CA, USA). Spots whose intensity was close to that of the local background or whose circularity or homogeneity was insufficient were excluded. Data were smoothed by local-weighted smoothing scatterplot (LOWESS) and the consistency of the flip-dye was verified. Spots whose value was between ±2 of the mean value of the distribution of spot values (slice analysis), or spots that were not present in the form of duplicates, were excluded. At the end, genes involved in metabolic pathways were considered to be differentially expressed if they displayed ≥2-fold changes and a *p*-value < 0.05. For genes coding for regulators, the differential expression was considered for ≥1.6-fold changes. For the *codY* gene, a specific wrapper-based gene selection technique was applied (bootstrap classification combined to variance analysis). Array data are available in the Supplementary Material Figure S2.

### 4.4. Model-Based Change Prediction for the Anteiso Fatty Acid Precurssor Overproduction

In order to predict by bioinformatic gene knock-out or gene overexpression, the reaction network model of the metabolic pathway of the branched chain amino acids leucine (Leu), valine (Val), and isoleucine (Ile) from threonine (Thr) and pyruvate (Pyr) in *B. subtilis* was used. This model was first presented in [27] when studying the overproduction of Leu, demonstrating that it is a crucial precursor of surfactin. This reaction network is shown in Figure S2. For the prediction of gene knockouts that may lead to Leu overproduction, a biocomputing reaction network tool was applied [49]. With the target out-Leu = ↑, the tool produced the predictions presented in Figure S3, where r = ⇓ stood for the prediction of the knockout of reaction r. The tool ran the prediction algorithm for reaction networks with partial kinetic information from [43], based on abstract interpretation. The idea was to abstract change between real values in the steady states to symbols, such as ↑ for an increase, Do for a decrease, and ⇓ for a decrease to 0. The possible changes for knockout predictions were reaction knockouts r = ⇓. The tool, however, could also predict increase inflows of a species S, that is in-S = ↑, and decreases of inflows in-S = ↓. Such predictions meant that changes of the experimental environment, or of adjacent reaction networks, may lead to the target.

**Figure 8:**
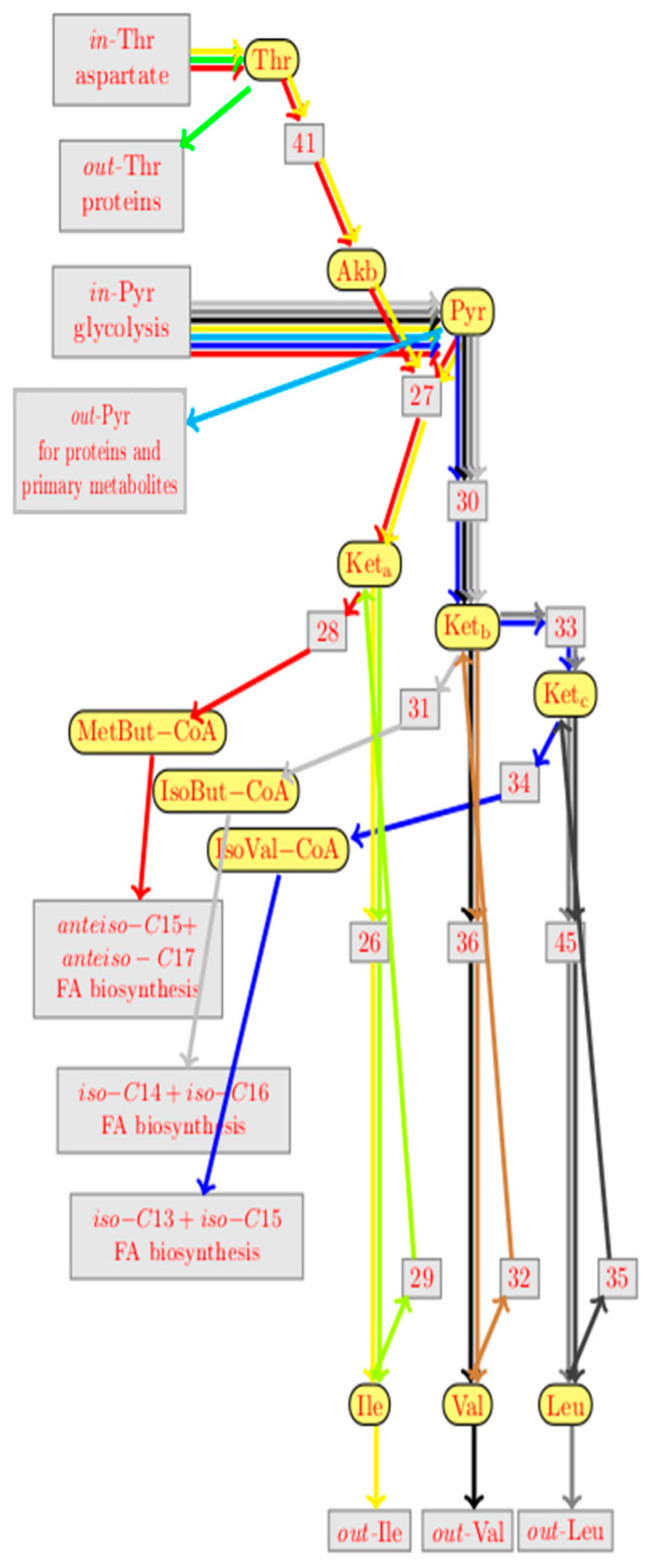
Elementary flux modes (EFMs) of the metabolic subnetwork of the reaction network in Figure S3. The dark gray flux mode show that inflow Pyr can be used to produce outflowing Leu. The black flux mode shows that Pyr can also be used to produce outflowing Val. Furthermore, the yellow flux mode shows that inflowing Pyr contributes to the production of Ile but only concomitantly with inflowing Thr.

Abstract interpretation enables local reasoning in the graph of the network graph. Local reasoning alone, however, is problematic for graphs with loops. In this case, it may lead to a large over-approximation, such as predicting changes which may not lead to the target. The original algorithm of the BioComputing’s tool, therefore, used heuristics based on rewritten rules for difference constraints [43] to reduce the over-approximation in the presence of loops. Meanwhile, an exact treatment of loops in the metabolic part of the network was developed [49], which relies on the usage of elementary flux modes (EFMs). For instance, the EFMs of the reaction network in Figure S3 are shown in Figure 8. The dark gray and black EFMs show that inflowing of Pyr can be used in the production of Leu and Val, respectively. The yellow EFMs show that Thr is needed for the production of Ile too, concomitantly with Pyr. The usage of EFMs helps to add nontrivial global reasoning power based on metabolic loops that is essential for precise predictions. Feedback loops through the regulatory control network, however, remain problematic, as will be analyzed hereafter.

To produce the *anteiso*-C17 mycosubtilin isoform, an increase in the outflow of MetBut-CoA into the FA biosynthesis of *anteiso*-C15 fatty acid is required. MetBut-CoA can, for instance, be produced from Ile. The reaction network in Figure S2 collapses MetBut-CoA with IsoBut-CoA and IsoVal-CoA into a single species called Acyl-CoA. So, in our previous model, one cannot distinguish the outflow of MetBut-CoA towards the wanted fatty acid chain, in particular *anteiso*-C15, from the outflows of IsoBut-CoA (coming from Val) toward the FA biosynthesis of *iso*-C14 and *iso*-C16 and the outflow of IsoVal-CoA (coming from Leu) to the FA bio-synthesis of *iso*-C13 and *iso*-C15. Therefore, the reaction network was modified in the present work to adapt the reaction network from Figure S2, so that it splits Acyl-CoA into species MetBut-CoA, IsoBut-CoA, and IsoVal-CoA. This is the first step leading from the reaction network presented in Figure S2 to that presented in Figure S3 (Figure 8).

Further improvements to the reaction network described below are shown in Figure S4. This figure contains some more important changes compared to Figure S2, not only the split of Acyl-CoA. In particular, new outflows for Thr and Pyr to the protein and other amino acids or primary metabolites synthesis were added. Biologically, this was justified since these amino acids can also be used in the production of proteins during gene expression elsewhere. This adds the green EFM moving inflowing Thr to its new outflow, and the light blue EFM moving inflowing Pyr to its new outflow (Figure 8). Change predictions that lead to over-activating these EFMs will turn out particularly relevant later on. Furthermore, some other simplifications were performed. First, the species glutamic acid (Glu) and oxoglutarate (OxoGlu) were removed. The main reason was that only carbon (C) was of interest, not azote (N). Second, the reactions 12 and 13 were revised since the changes of 12 and 13 were the same biologically. This led us to the reactions 62 and 63. Finally, an algorithm was also improved to not only predict gene knockouts but also gene overexpression. For this, however, the biocomputing reaction network tool was extended with the ability to overexpress candidate genes.

After all these modifications, the use of the BioComputing’s reaction network tool on the reaction network did not work well the first time, for several reasons. The first problem was that the addition of the outflows out-Thr and out-Pyr increased the computation time, so that only few predictions could be observed after some hours. Some results could be only obtained in reasonable computation time when the outflows were removed again, as presented in Figure S2. The second problem was that a large number of predictions was made, as shown in Figure S3, some of which do not seem to be relevant and cannot be justified biologically, especially as most of them are common to the previous predictions made with Leu = ↑ as the target [27]. As a result, it was difficult to determine whether these were false positives or not from a biological or bioinformatic point of view. It turned out that the unexpected predictions were spontaneous over approximations. It was then noticed that these over-approximations were related to feedback loops through network regulation. However, with the currently available algorithms, it was not possible to dispose of these over approximations. Therefore, a decision was made to remove the regulatory feedback loops, even if they were biologically justified, resulting in the reaction network presented in Figure S4.

The refined reaction network was then used to predict the overproduction of odd *anteiso* fatty acids (Figure 5). When these predictions were launched, a final problem was detected, related to the very high computation time of the solver used by the BioComputing’s reaction network tool. This raised serious difficulties when adding the outflows out-Thr and out-Pyr in particular, as presented above. The reason for this problem was linked to the search strategy chosen by the solver. Once the search strategy was improved, single changes in Figure 5 were predicted in a few seconds.

### 4.5. Lipopeptide HPLC Analysis

Samples of *B. subtilis* cultures were centrifuged at 10,000× *g* for 10 min. A volume of 1 mL of the supernatant was purified through C18 Maxi-Clean cartridges 1 g (Alltech, Deerfield, IL, USA). The column was washed with 8 mL of water. The lipopeptides were then eluted with 8 mL of 100% methanol (HPLC grade, Acros Organics, Geel, Belgium). The extract was dried before dissolution in 200 µL of methanol. The sample was then injected and analyzed by high-performance liquid chromatography (Online Degaser, 717 Autosampler, 660S Controller, 626 Pump, 996 PhotoDiodeArray, Waters Corp., Milford, MA, USA) using a C18 column (5 µm, 250 × 4.6 mm, 218 TP, VYDAC). The mycosubtilins were separated with an acetonitrile/water/trifluoroacetic acid solvent, 40:60:0.1, *v*/*v*/*v*. The flow rate was 1 mL/min and the detection wavelength was 214 nm. Purified iturin A and surfactin A, used as standards, were from Sigma (Sigma-Aldrich, Saint Louis, MI, USA). The retention time and second derivative of the absorption spectrum between 200 and 400 nm (Diode Array PDA 996, Waters) were used to identify the eluted molecules (Millenium 32 Software, Waters).

### 4.6. Lipopeptide Preparation

A volume of 10 mL of the culture supernatants was purified through C18 Maxi-Clean 10 g cartridges (Alltech). Compared to the lipopeptide HPLC analysis protocol, all the volumes used were adapted. A sample of 100 μL of the lipopeptide mixture concentrated at 250 mg/L was charged in the HPLC (Online Degaser, 717 Autosampler, 660S Controller, 626 Pump, 996 PhotoDiodeArray, Waters Corp., Milford, MA, USA). The column used was a C18 (5 μm, 300 × 10 mm, ACE), working at a flow rate of 3 mL/min. The mycosubtilins were separated with an acetonitrile–water–trifluoroacetic acid solvent, 35:65:0.1, *v*/*v*/*v* for 54 min, followed by a 6 min rinsing step with acetonitrile–trifluoroacetic acid solvent, 100:0.1, *v*/*v*. As for lipopeptides analysis, the retention time and second derivative of the absorption spectrum between 200 and 400 nm were used to identify the eluted molecules (Millenium 32 Software, Waters).

#### 4.6.1. Lipopeptide Tandem Mass Spectrometry MS-MS Analysis

The different isoforms of mycosubtilin produced by *B. subtilis* BBG100 were determined by analyzing the purified samples with tandem mass spectrometry (MS-MS), electrospray, and an ion trap. Measurements were made by direct infusion of the open peptide after treatment with N-bromosuccinimide, as described previously [9,60].

#### 4.6.2. Determination of the Mycosubtilin Isoforms by GC

The identification of mycosubtilin isoforms was completed with performing GC. The protocol used for hydrolysis of mycosubtilin was the one for iturinic compounds [33]. Briefly, 1 mg of lipopeptide was hydrolyzed in the presence of 12 M HCl/methanol (30/70) during 15 h at 150 °C. Methanol was evaporated and fatty acids were extracted with chloroform. The extract was washed twice with water milliQ. Chloroform was evaporated and the fatty acids were suspended in 100% methanol. The fatty acid methyl esters (FAMEs) of the samples and the reference compounds were prepared by boron trifluoride-catalyzed methylation (AOCS, 1998) and analyzed by gas chromatography (GC). The analysis of the MEFAs in hexane was performed with a GC system (HP6890, Agilent Technologies Inc., Santa Clara, CA, USA) equipped with a split–splitless injector and a flame ionization detector. An INNOWAX capillary column (Agilent, ref.19091N-133; length, 30 m; diameter, 0.25 mm; 0.25 m film) was used to separate the MEFAs. The GC conditions were as follows: injector temperature at 250 °C and detector temperature at 250 °C. The carrier gas was helium at a flow rate of 2.4 mL/min. The temperature program is 10 min at 50 °C, 30 °C/min to 150 °C; 4 °C/min to 240 °C; and 10 min at 240 °C. The fatty acids were identified by GC by an injection of Supelco (ref.47885-U) and Larodan standards for *iso* and *anteiso* fatty acids (ref.11-1514-7 and 11-1614-7).

### 4.7. Cellular Fatty Acid Analysis

The cells were harvested from the 100 mL cultures and centrifugated at 10,000× *g* for 10 min at 4 °C. The pellets were washed twice with sterile distilled water and transferred into extraction screw-cap tubes. Bacteria were subsequently submitted to saponification and methylation, and their fatty acid methyl esters were extracted, as described previously [61]. Analysis of fatty acid methyl esters was carried out by gas chromatography performed on a Shimadzu GC14A gas chromatograph (Courtaboeuf, France) equipped with a 30 m × 0.25 mm BPX5 (SGE, Villeneuve St George, France) capillary column, as previously described [62].

### 4.8. Bacterial Dry Weight Analysis

The bacterial dry weight was determined after drying for 48 h at 110 °C in a washed pellet of a 10 mL sample. The optical density at 600 nm was read with a spectrophotometer SECOMAN Prim (SECOMAN, Domont, France).

### 4.9. Determination of Minimal Inhibitory Concentrations

Antifungal activity tests against *A. niger* were performed by successive half-dilutions of various solutions of mycosubtilin isoforms (*iso*-C16, *n*-C16, *anteiso*-C17 and *iso*-C17) in 96-well microplates containing 200 µL of culture medium (glucose, 40 g/L; peptone, 10 g/L; yeast extract, 2 g/L) [13]. A standard mycosubtilin mixture was also tested, composed of *iso*-C16 (26%), Gln3 C17 (1%), *n*-C16 (2%), *anteiso*-C17 (45%), and *iso*-C17 (23%). The inoculation of the two microorganisms was realized at an optical density of 0.55 followed by an incubation at 28 °C and 280 rpm. The minimum inhibitory concentration (MIC) was determined after 24 h of incubation.

## 5. Conclusions

In this work, we implemented a metabolic engineering strategy based on two approaches in parallel (transcriptomics and bioinformatics) to target genetic modifications, allowing the selective production of mycosubtilin isoforms, and more particularly, the C17-*anteiso* isoform. Thanks to a major refinement of the model of the previously developed BCA metabolic pathway, we were able to predict new gene knock-out or gene overexpression targets, tested of which were tested *in vivo*. Overexpression of the *ilvA* gene showed a significant impact on the production of the desired mycosubtilin isoform, while *codY* knockout showed more contrasting results. This work allowed us to refine the prediction tool, making it more reliable, notably by simplifying certain reactions and removing feedback loops. However, from a bioinformatics point of view, further research is needed to obtain equally reliable predictions, taking into account the feedback loops. In addition, the other predictions shown in this work represent a gold mine of information for future optimization work.

## Figures and Tables

**Table 1:** MIC determination of the mycosubtilin mixture and mycosubtilin-purified isoforms expressed in µM. Experiments were performed in triplicate.

Pathogens Strains	Mixture	*iso*-C16	*n*-C16	*anteiso*-C17	*iso*-C17	Reference
*Botrytis cinerea*	8	32	16	8	16	[9]
*Aspergillus niger*	8	32	16	8	16	This work
*Candida albicans*	8	>32	8	32	16	[7]

**Table 2:** Plasmid and strains used in this study.

Strains		Genotype/Phenotype	Reference or Source
*E. coli*	JM 109	*recA*1, *endA*1, *gyrA*96, *thi*, *hsdR*17, *relA*1, sup*E*44, Δ(*lac*-*proAB*), [F′, *traD*36, *proAB*, *lacI*/*lacZ*ΔM15]	Promega
*B. subtilis*	ATCC 6633	Wild-type strain/Myc+, Srf+	Laboratory stock
	BBG100	ATCC 6633 *PrepU neo*:*:myc*/Myc+++; Srf+, Nm^R^	Laboratory stock [35]
	RFB102	ATCC 6633 *amyE*::P*spac-comK-spc/*Spc^R^	Laboratory stock [7]
	BBG133	RFB102 *PrepU-neo::ilvA/*Nm^R^	This work
	BV12I37	ATCC 6633 Δ*codY, cat kan/*Cm^R^, Km^R^	[48]
**Plasmids**			
	pGEM-T Easy	Cloning vector/Ap^R^	Promega
	pBEST501	pUC9:: *PrepU-neo/*Nm^R^	Laboratory stock
	pBG142	pGEM-T Easy *lacZ::ilvA*/Ap^R^	This work
	pBG154	pGEM-T Easy *lacZ::PrepU-neo::ilvA*/Ap^R^, Nm^R^	This work

Ap^R^: ampicillin resistance; Nm^R^: neomycin resistance, Spc^R^: spectinomycin resistance, Cm^R^: chloramphenicol resistance, Km^R^: kanamycin resistance.

## Data Availability

The data presented in this study are available in article or Supplementary Material.

## Supplementary Materials

The following supporting information can be downloaded at: https://www.mdpi.com/article/10.3390/metabo12020107/s1, Figure S1: HPLC chromatogram of the mixture of mycosubtilin produced by *B. subtilis* ATCC 6633. Mycosubtilins were identified according to their retention time: mycosubtilin *iso*-C16 (34.439 min), mycosubtilin *n*-C16 (36.420 min), mycosubtilin *anteiso*-C17 (42.703 min) and mycosubtilin *iso*-C17 (43.812 min); Figure S2: Original model of Branched Chain Amino Acid metabolic pathway developed for Leu overproduction and proposed by Coutte et al. (2015) [27]; Figure S3: Original model of Branched Chain Amino Acid metabolic pathway developed for Leu overproduction [27] with only knock-out prediction indicated in dark orange and represented by ⇓; Figure S4: New reaction network of Branched Chain Amino Acid metabolic pathway developed for outflow of MetBut-CoA to the FA biosynthesis of *anteiso*-C15 and *anteiso*-C17 fatty acid chains. This picture can be compared with the Figure S3 to see evolution in the model, Table S1: Supplementary data of raw microarray results.
