# Supplementary material for: Bioinformatics Modelling and Metabolic Engineering of the Branched Chain Amino Acid Pathway for Specific Production of Mycosubtilin Isoforms in Bacillus subtilis
Source: Metabolites. 2022 Jan 24;12(2):107. doi: 10.3390/metabo12020107 (PMC8877110; doi:10.3390/metabo12020107)
Supplement: Supplementary file 1 [file metabolites-12-00107-s001.zip › Supplementary Figures.pdf]

metabolites

MDPI

Supplementary Data

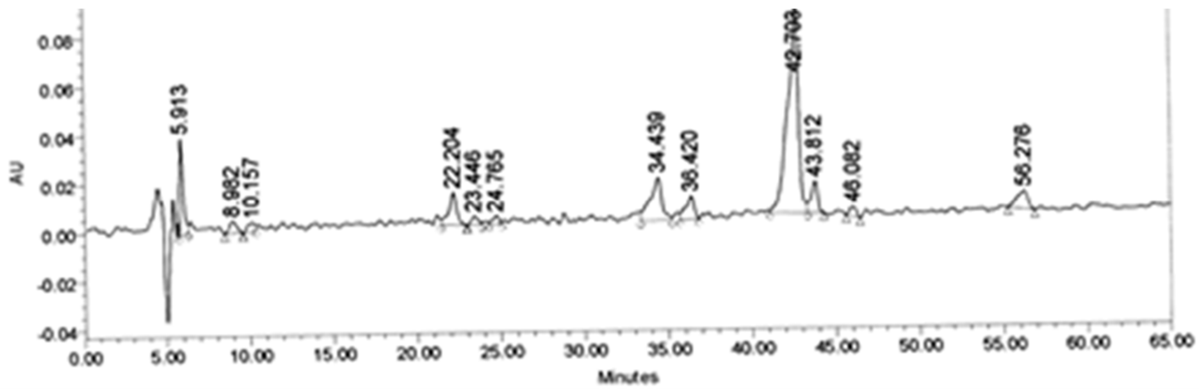

Figure S1. HPLC chromatogram of the mixture of mycosubtilin produced by *B. subtilis* ATCC 6633. Mycosubtilins were identified according to their retention time: mycosubtilin *iso*-C16 (34.439 min), mycosubtilin *n*-C16 (36.420 min), mycosubtilin *anteiso*-C17 (42.703 min) and mycosubtilin *iso*-C17 (43.812 min).

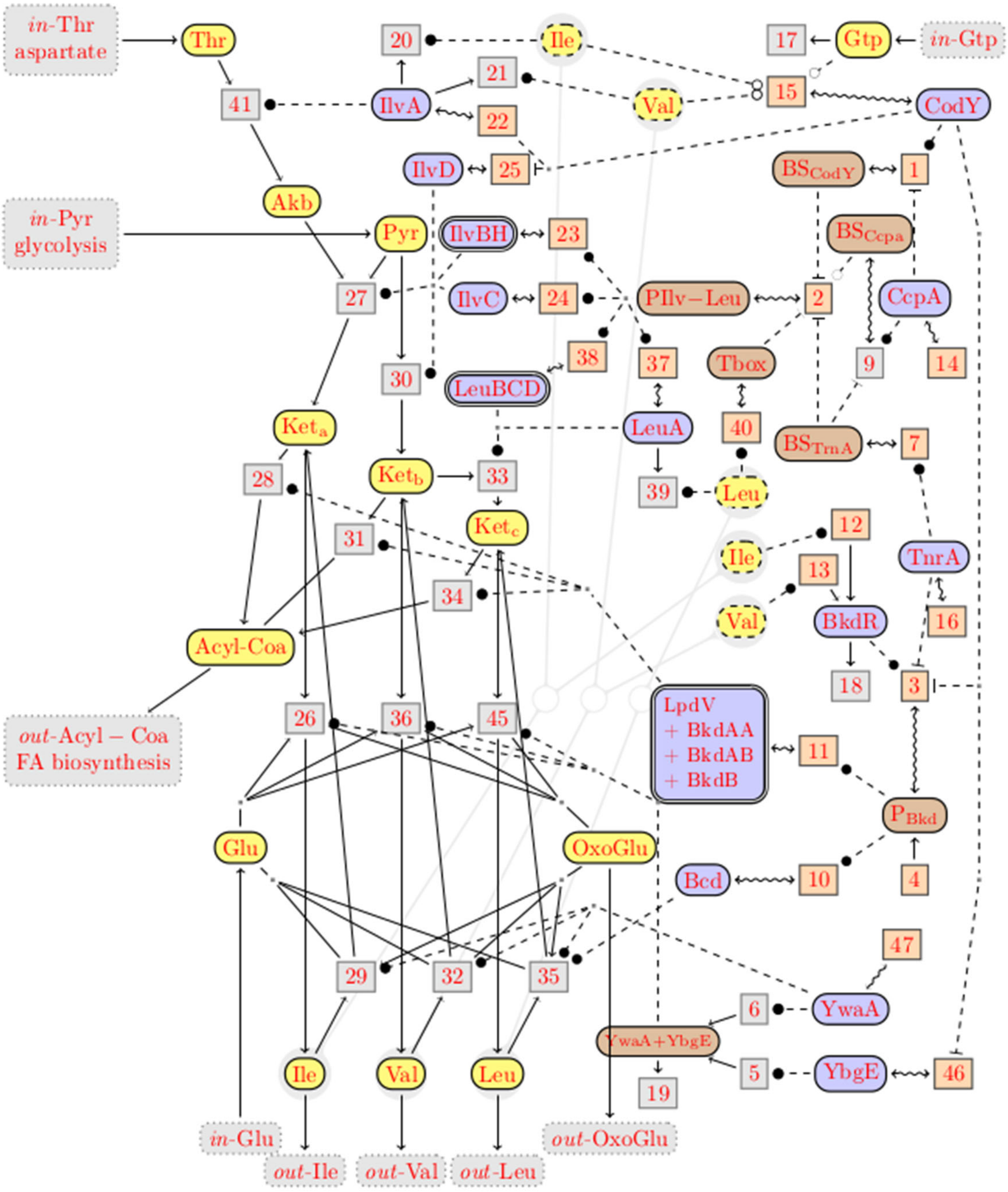

Figure S2. Original model of Branched Chain Amino Acid metabolic pathway developed for Leu overproduction and proposed by Coutte et al., 2015 [27].

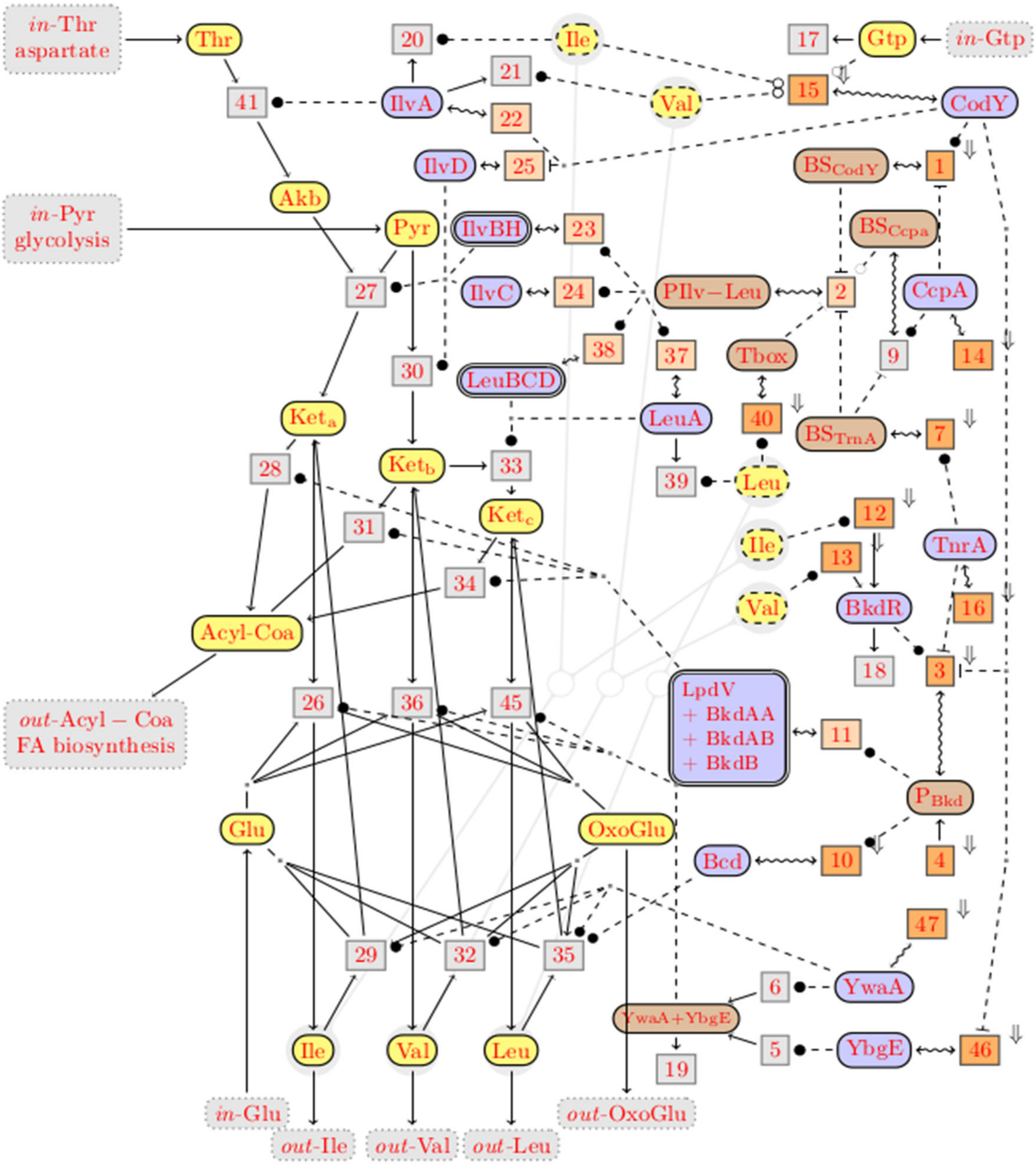

Figure S3. Original model of Branched Chain Amino Acid metabolic pathway developed for Leu overproduction [27] with only knock-out prediction indicated in dark orange and represented by ⇓.

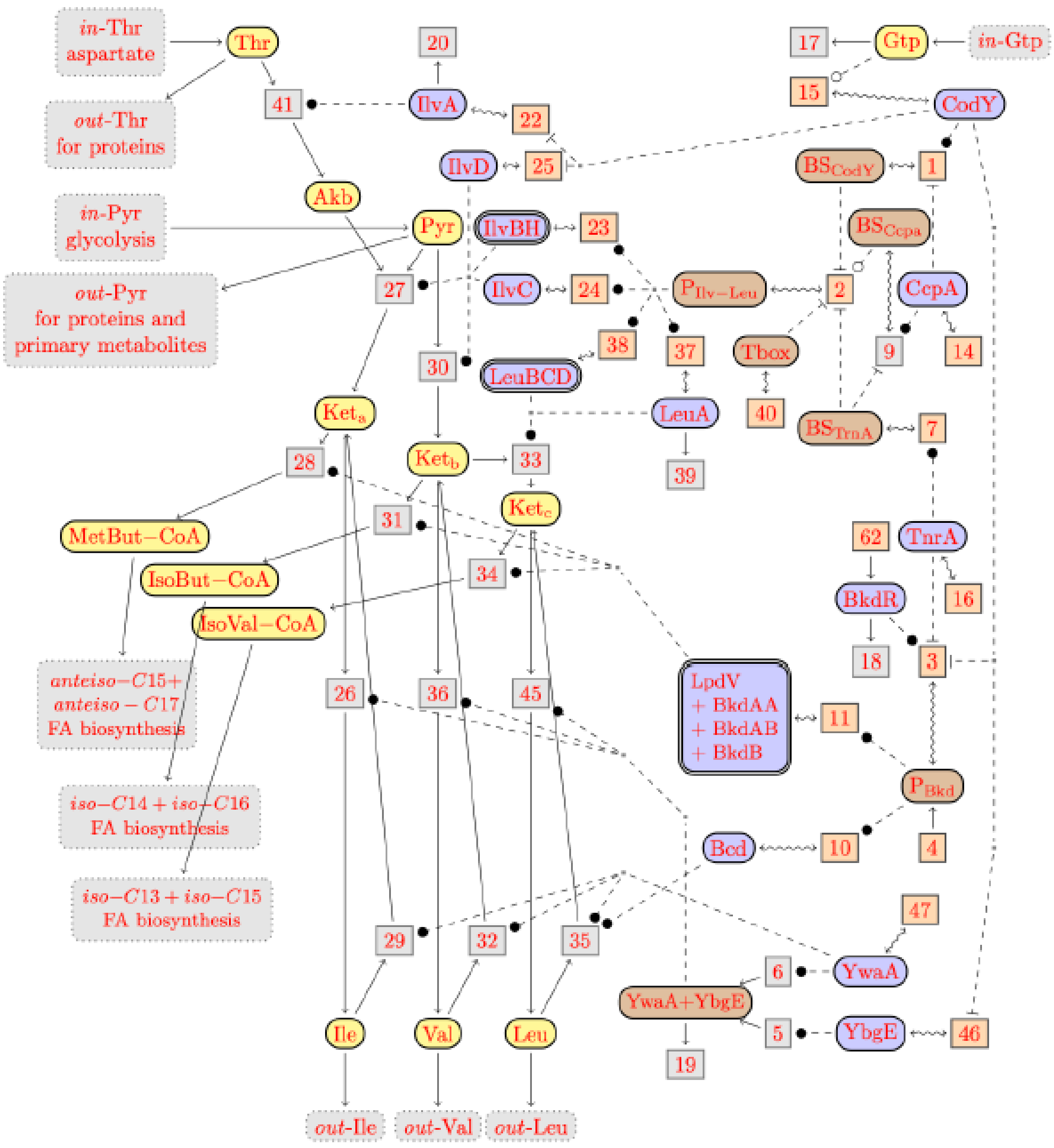

Figure S4. New reaction network of Branched Chain Amino Acid metabolic pathway developed for outflow of MetBut-CoA to the FA biosynthesis of *anteiso*-C15 and *anteiso*-C17 fatty acid chains. This picture can be compared with the Figure S3 to see evolution in the model.

Excel file. Supplementary data of raw microarray results
